# Outcomes of Dialysis Modality Switch: A Matched Cohort Analysis from a National Renal Replacement Therapy Registry, 2010–2022

**DOI:** 10.3390/jcm15103948

**Published:** 2026-05-20

**Authors:** Chen Namimi-Halevi, Margarita Kunin, Michal Bromberg, Rita Dichtiar, Pazit Beckerman

**Affiliations:** 1 Israel Center for Disease Control, Israel Ministry of Health, Ramat Gan 52621, Israel; michal.bromberg@moh.gov.il (M.B.); rita.dichtiar@moh.gov.il (R.D.); 2 The Institute of Nephrology and Hypertension, Sheba Medical Center, Tel-Hashomer, Ramat Gan 52621, Israel; margarita.kunin@sheba.health.gov.il (M.K.); pazit.beckerman@sheba.health.gov.il (P.B.); 3 School of Medicine, Gray Faculty of Medical and Health Sciences, Tel-Aviv University, Tel Aviv 6997801, Israel; 4 Department of Epidemiology and Preventive Medicine, School of Public Health, Gray Faculty of Medical and Health Sciences, Tel-Aviv University, Tel Aviv 6997801, Israel

**Keywords:** dialysis modality switching, hemodialysis, peritoneal dialysis, mortality, matched cohort, end-stage renal disease, cause of death, national registry

## Abstract

**Background/Objectives:** Evidence on mortality after dialysis modality switch is inconsistent and may vary by timing and type of switch. This study examined associations between modality switch and short- and long-term mortality and compared cause-of-death patterns across groups. **Methods:** This matched historical cohort study used the Israeli National Renal Replacement Therapy Registry (2010–2022). Adult dialysis patients with a modality switch were matched 1:1 to non-switchers by age, initial modality, and survival to switch time. Follow-up started at the switch date or matched index date. Outcomes were all-cause mortality at 3 months, 6 months, 1 year, and 2 years. Conditional Cox models estimated adjusted hazard ratios overall, by initial modality, and by early (≤180 days) or late switch. Among 2-year decedents, logistic regressions compared primary and multiple cause-of-death distributions. **Results:** The cohort included 892 switchers and 892 non-switchers (median age 65.0 years; 66.1% males; 64.2% peritoneal dialysis [PD]-first). Among PD-first late switchers, mortality hazards were lower across follow-up landmarks, with the greatest reduction at 2 years (HR 0.421; 95%CI: 0.306–0.579), whereas hemodialysis (HD)-first early switchers had higher mortality, with borderline significance (HR 1.614; 95%CI: 0.951–2.740). Among 591 deaths within 2 years, primary cause-of-death distributions were similar; for multiple causes, early switchers showed higher adjusted odds of heart disease, whereas late HD-initiated switchers had higher adjusted odds of renal disease. **Conclusions:** Mortality after modality switch varies by transition direction and timing. Specifically, a late PD-to-HD transition was associated with lower long-term mortality. Further studies are needed to clarify the roles of clinical/transition-related factors.

## 1. Introduction

Hemodialysis (HD) and peritoneal dialysis (PD) are the two principal modalities of life-sustaining renal replacement therapy (RRT) for patients with end-stage renal disease (ESRD) [1]. In contemporary observational cohorts, the survival difference between initiating PD versus HD appears to have narrowed substantially [2,3,4]. Earlier studies often reported an apparent early survival advantage among PD initiators that attenuated with longer follow-up, whereas more recent analyses employing more robust adjustment strategies and reflecting modern dialysis practices have generally suggested broadly comparable mortality between modalities [2,5].

However, in clinical practice, patients may experience one or more modality transitions over time because of technique failure, complications, evolving comorbidity, vascular access considerations, or patient preference [6,7,8]. In many high-income settings, PD-to-HD transfer rates are often reported at 10–20% per year [3,9], while HD-to-PD transfer is less common (<5% of HD starters in previous reports) [10,11].

Current evidence regarding the mortality risk associated with these transitions exhibits considerable heterogeneity. For patients transitioning from PD to HD, research identifies the immediate short-term period as a high-risk interval characterized by peak mortality rates [12,13]. However, long-term assessments, particularly within integrated care systems, suggest that patients who successfully navigate this transition can achieve survival outcomes comparable to those maintained on a single modality [14]. Studies examining the less frequent transition from HD to PD have yielded inconsistent findings as well [3,10,15].

Heterogeneity also exists in the association between transition timing and subsequent outcomes. While some evidence links prolonged treatment duration on the preceding modality to higher risks of mortality [10,12], other analyses indicate that the timing of the switch may not serve as an independent predictor of long-term survival [13,15].

Despite the clinical frequency and potential prognostic importance of a modality switch, the research base remains limited. There is a particular need for additional evidence derived from large, contemporary national registry datasets that can capture complete RRT trajectories, enable robust adjustment for confounding, and support adequately powered evaluations of outcomes across distinct transition pathways and timing [4,16,17,18].

Therefore, this study examines the association between dialysis modality switch and both short-term and long-term mortality among patients receiving dialysis, using data from a national registry. The analysis is stratified by transition direction (PD-to-HD versus HD-to-PD) and by transition timing (early versus late) to better characterize heterogeneity in risk across clinically distinct switch patterns. In addition, differences in causes of death between patients who switched modality and those who did not are evaluated to provide complementary insight.

## 2. Methods

### 2.1. Study Design and Data Sources

This study employed a matched historical cohort design using data from the Israeli National Renal Replacement Therapy Registry (INRRTR), managed by the Israel Center for Disease Control under the Ministry of Health [19]. The registry includes all Israeli patients receiving any form of RRT (HD, PD, or kidney transplantation) and is based on mandatory reporting by dialysis facilities, health funds, and the National Transplant Center.

### 2.2. Study Population and Eligibility Criteria

The study population comprised all adult ESRD patients aged ≥18 years who initiated dialysis therapy in Israel between 1 January 2010 and 31 December 2022.

From this population (*n* = 20,533), all treatment transitions were identified (*n* = 1139) and evaluated for eligibility according to the 30-day rule, which ensured valid treatment periods: each dialysis modality had to last at least 30 consecutive days to be considered a distinct treatment episode, as previously described [20].

If a patient switched modalities within fewer than 30 days, that short episode was deemed non-qualifying and was disregarded. When a patient initiated dialysis with one modality and switched after <30 days, the first treatment was excluded, and the second was considered their true starting modality. If a patient remained on the first modality for ≥30 days and then switched, that first episode was valid. If the second treatment lasted <30 days and was followed by another switch, the second episode was ignored, creating a continuous sequence between the first and third modalities.

After applying the 30-day treatment-episode algorithm, switchers were defined using the following criteria:

Inclusion criteria:

- Patients with at least one qualifying switch (i.e., ≥30 days in each preceding modality) were classified as switchers.
- For patients with more than one qualifying modality switch, only the first qualifying switch was used to define exposure, matching, and follow-up.

Exclusion criteria:

- Patients whose only transition was non-qualifying (a single switch lasting <30 days) were excluded, as they could not be consistently categorized as either switchers or non-switchers (*n* = 72).
- Patients who switched modality after 31 December 2022 were excluded (*n* = 115).
- Patients whose modality switch occurred after kidney transplantation or after discontinuation of dialysis for other reasons (*n* = 15).

Overall, 198 switchers were excluded. The exclusion counts are not mutually exclusive, as some patients met more than one criterion.

**Figure 1:**
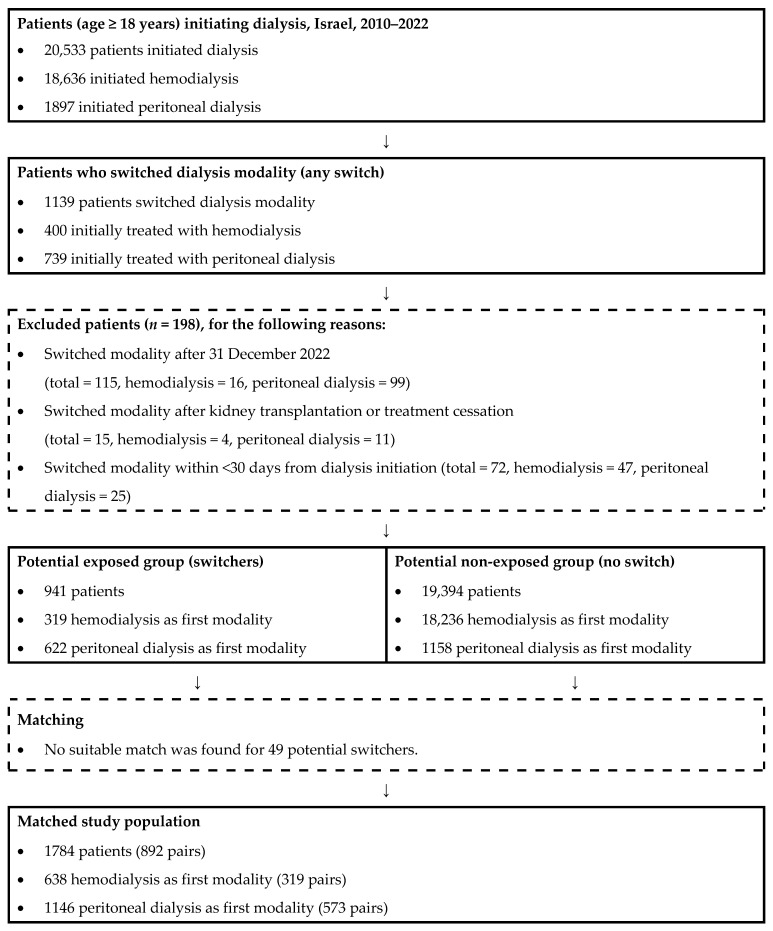
Flowchart of Study Participants.

For each switcher, one non-switcher control was matched (1:1 ratio) based on: (A) age (±5 years); (B) initial treatment modality (HD or PD); (C) to ensure comparable follow-up opportunities, the matched control was required to survive at least the same duration as the time elapsed from the switcher’s treatment initiation until the date of treatment change. Matching was performed randomly when multiple eligible controls were available. Each control was used only once (Figure 1).

### 2.3. Exposure Definition

The primary exposure variable was treatment switch, defined as a change in dialysis modality that met the minimum 30-day duration criterion described above. Patients were classified as switchers if they had at least one such valid transition during the follow-up period and as non-switchers otherwise.

### 2.4. Outcome Variables

#### 2.4.1. Mortality

The primary outcomes were 3-month, 6-month, 1-year, and 2-year all-cause mortality following the index date. For switchers, follow-up began on the date of the first qualifying modality switch. For non-switchers, follow-up began on their dialysis initiation date plus the number of days elapsed between the matched switcher’s dialysis initiation and modality change, ensuring temporal alignment of dialysis vintage within matched pairs.

We chose this approach because the main objective was to compare post-transition outcomes among patients who had already reached a clinically defined transition time, rather than to model the entire dialysis course as a continuous time-varying exposure process.

Censoring events included kidney transplantation, cessation of dialysis therapy, or end of follow-up (3 months, 6 months, 1 year, or 2 years, depending on the analysis). In sensitivity analyses, additional censoring was applied at the time of a subsequent valid modality change.

Mortality data were obtained from the Ministry of Interior, transplantation dates from the National Transplant Center, and dialysis cessation dates from the INRRTR.

#### 2.4.2. Causes of Death Data and Classification

For patients who died within two years of the index date, causes of death were obtained from the Central Bureau of Statistics (CBS) mortality files and classified into five mutually exclusive categories based on the causes of death coded according to the International Classification of Diseases (ICD), 9th and 10th Revisions. Causes of death were analyzed in two ways: (1) by underlying cause of death, classified into five mutually exclusive categories; and (2) by multiple-cause-of-death coding, in which each category was assessed separately as present or absent among contributory causes listed on the death certificate. The analysis was conducted at the group level rather than within matched pairs, comparing switchers and non-switchers as two cohorts.

It should be noted that these analyses compare the distribution of causes of death among decedents within two years (case–case) and do not estimate cause-specific mortality risks/hazards in the full cohort.

Heart disease included ICD-10 codes I00–I09, I11, I13, and I20–I51, and ICD-9 codes 390–398, 402, 404, and 410–429. Infectious diseases included ICD-10 codes A00–A32, A34–A99, and B00–B99, and ICD-9 codes 001–134 and 136–139. Cerebrovascular disease (CVA) included ICD-10 codes I60–I69 and G45, and ICD-9 codes 430–438. Renal disease included ICD-10 codes N00–N12, N17–N19, and N25–N29, and ICD-9 codes 580–589. All remaining causes of death were grouped under a composite “Other” category.

### 2.5. Additional Variables

Additional variables were derived from the INRRTR and other data sources, including the following:

- **Age (years)** at dialysis initiation.
- **Sex:** Male/female.
- **Population group:** Jews and others vs. Arabs. The “Others” category includes individuals who are neither Jewish nor Arab, such as non-Arab Christians, members of other religions, and individuals without a religious classification, following the official CBS definition [21].
- **Socioeconomic status (SES):** Derived by cross-referencing patients’ residential Statistical Geographic Areas (SGAs) with the Points Location Intelligence (PLI) index [22], which is based on the CBS socioeconomic index. The CBS index integrates demographic composition, education level, living standards, employment, and pension data, and the PLI continuously updates these data to enhance local accuracy. SES was classified into low (1–3), medium (4–6), and high (7–10) categories, representing the decile ranges of the national distribution.
- **Peripherality:** Calculated according to the CBS Peripherality Index, which quantifies the degree of a locality’s accessibility relative to other population centers in Israel [23]. The index is a weighted sum of two components: (A) a potential accessibility index, representing the proximity of the locality to all other local authorities weighted by their population sizes; and (B) proximity to the border of the Tel Aviv District, which reflects geographic distance from Israel’s main economic and business hub. Based on the CBS classification, peripherality was grouped into peripheral (1–3), intermediate (4–6), and central (7–10) categories.
- **Religious orthodoxy:** Derived by cross-referencing patients’ residential SGAs with the orthodoxy PLI index, categorized according to residential concentration of ultra-Orthodox Jewish population (low or high levels) [22].
- **First dialysis modality:** Categorized as HD or PD, as reported by the first treating dialysis facility.
- **Incident-year cohort:** Categorized as 2010–2013, 2014–2017, or 2018–2022.
- **Facility type:** Categorized as *hospital-based* or *community-based* according to the setting of the patient’s initial dialysis treatment. Because PD initiation in Israel occurred exclusively in hospital-based settings during the study period, this variable differentiated only among HD initiators.
- **Primary kidney disease:** As reported by the dialysis centers using diagnostic codes from the European Dialysis and Transplant Association (EDTA), officially known as the *European Renal Association—European Dialysis and Transplant Association (ERA-EDTA)* coding system [24]. This system classifies the underlying etiology of kidney failure at the time of treatment initiation. Diagnoses were grouped into five major categories: (A) diabetes mellitus, (B) hypertension and renal vascular disease, (C) glomerulonephritis, (D) other, and (E) unknown/missing.
- **Time to modality switch:** Categorized as early (≤180 days from initiation of the first dialysis modality to the date of modality change) or late (>180 days). The matched non-switcher control was assigned the same time-to-switch category as the corresponding switcher. A sensitivity analysis incorporated an alternative 90-day cut-off threshold [25].

### 2.6. Power Analysis

No formal a priori sample size calculation was conducted, because this was a nationwide registry-based study including all eligible switchers and their matched non-switcher controls. Nevertheless, we conducted an event-based power assessment for the primary 2-year mortality analysis using Schoenfeld’s approximation for Cox proportional hazards models. Assuming a two-sided α of 0.05 and equal allocation between switchers and non-switchers, approximately 379 events would be required to detect a hazard ratio of 0.75 or 1.33 with 80% power. Given the observed 2-year event proportion of 33.1%, this would correspond to an approximate total sample size of 1145 patients. These effect sizes are conservative relative to several previously reported associations in studies of dialysis modality transitions [14,15,26,27]. The present matched cohort included 1784 patients and 591 deaths within 2 years, providing approximately 94% power for this effect size. Thus, the study appeared adequately powered to detect moderate overall associations with 2-year mortality, although power was more limited in smaller stratified analyses.

### 2.7. Statistical Analysis

All analyses were conducted using SAS software (version 9.4, SAS Institute Inc., Cary, NC, USA). Descriptive statistics were used to summarize patient characteristics. Continuous variables were expressed as medians (interquartile ranges), and categorical variables as frequencies (percentages). For matched comparisons between groups, the Wilcoxon signed-rank test was used for continuous variables, and McNemar’s test/Bowker’s test was applied for categorical variables. For unmatched comparisons (causes-of-death analysis), Mann–Whitney and Chi-square tests were applied for continuous and categorical variables, respectively.

Kaplan–Meier curves and log-rank tests were used descriptively to compare unadjusted survival patterns between switchers and non-switchers at the group level. The matched-pair structure was not retained in these descriptive curves.

Mortality Hazard Analysis: For multivariable analyses, regression models were constructed while accounting for the matched study design and adjusting for potential confounders. Conditional Cox proportional hazards models were applied for mortality outcomes. Analyses were performed in the overall study population and were additionally stratified by initial treatment modality and by time to modality switch. Hazard ratios (HRs) and 95% confidence intervals (CIs) were estimated for switchers compared with non-switchers.

The multivariable models were adjusted for core sociodemographic variables selected a priori, irrespective of the results of the unadjusted analyses, provided that these variables were not used for matching. Due to substantial collinearity between population group and SES (Spearman’s r = 0.54, *p* < 0.001), population group was retained in the models in preference to the ecological SES variable. Additional variables were included in the adjusted models if they showed an association at *p* < 0.1 in the univariate analyses.

Observations with missing values in covariates were excluded from the regression models, as the proportion of missingness was negligible. An exception was primary kidney disease, for which the proportion of unknown or missing values was considerable (17.1%); therefore, a separate “missing” category was included in the statistical models.

Sensitivity analyses were conducted using an alternative censoring approach and an alternative cut-off for early switch, as described above.

Cause of Death Analysis: Since these analyses were restricted to decedents within a fixed follow-up window and did not involve time-to-event information, binary logistic regression was used rather than time-to-event or conditional models. The analyses were conducted both for the underlying (primary) cause of death and for multiple causes of death, as described above. For each cause-of-death category, the outcome was defined as death from that specific cause versus death from all other causes. Unadjusted models included switch status as the sole independent variable, while adjusted models accounted for baseline sociodemographic and clinical covariates, with adjustment performed according to the principles specified above.

The regressions were also stratified by the initial modality and by timing of the switch; in the latter analysis, the non-switcher group remained unchanged, and stratification was performed only among the switchers.

Odds ratios (ORs) and 95% CIs were estimated for switchers compared with non-switchers.

All statistical tests were two-tailed, and *p*-values < 0.05 were considered statistically significant, unless otherwise mentioned.

### 2.8. Ethics

The study was approved by the Ethics Committee of the Israeli Ministry of Health (approval number: MOH-119-2024). As it was based on de-identified registry data, no individual informed consent was required. Data were fully anonymized, and no identifying details (such as names, ID numbers, or full addresses) were available to the investigators.

## 3. Results

### 3.1. Patients’ Characteristics

The sample included 1784 matched participants initiating dialysis in 2010–2022, comprising 892 switchers and 892 non-switchers (Table 1). Overall, the median age was 65.0 years (Q1, Q3: 55.6, 73.2), two-thirds were male, and 78.0% were Jews/Others. SES was low in 20.5% of patients, orthodoxy level was predominantly low, and approximately 62% resided in central areas. Among switchers, the median age was similar (64.9 years; Q1, Q3: 55.7, 73.4); 67.8% were male, 77.7% were Jews/Others, 20.8% had low SES, 60.6% lived in central areas, and over 95% had a low orthodoxy level. Most switchers initiated PD (64.2%). The median time until switching was 324 days (Q1, Q3: 120, 712); 34.3% were early switchers and 65.7% were late switchers.

Across groups, distributions of sex, population group, SES, and peripherality were broadly similar (all *p* > 0.05). The most notable differences were temporal and facility-related: compared to non-switchers, switchers were more common in earlier incident-year cohorts (2010–2013: 31.4% vs. 24.1%) and less common in 2018–2022 (31.3% vs. 40.5%) and were more frequently treated in hospital facilities (among all participants, 92.7% vs. 87.8%; and among hemodialysis initiators, 79.6% vs. 65.8%) (*p* < 0.001 for both). The distribution of the primary renal disease also differed between groups (*p* = 0.039; e.g., diabetes mellitus was the primary renal disease in 47.1% vs. 42.8% of switchers and non-switchers, respectively).

### 3.2. Survival Probabilities by Modality Switch Status

Short-term survival differences between switchers and non-switchers were modest, with clearer separation at longer follow-up: switchers had higher 1-year survival than non-switchers (0.827 vs. 0.785; *p* = 0.023) and higher 2-year survival (0.698 vs. 0.596; *p* < 0.001).

These patterns varied by initial modality and switch timing. Among patients initiating PD, switchers had higher survival at each landmark, with pronounced separation by 2 years (0.725 vs. 0.519; *p* < 0.001). In contrast, among those initiating HD, survival estimates were broadly similar between groups throughout follow-up. Late switch was associated with higher survival than non-switch, most notably at 1 and 2 years (2-year survival: 0.715 vs. 0.553; *p* < 0.001), whereas early switch showed survival similar to non-switch throughout.

In the combined strata, among patients initiating PD who switched late, survival was consistently higher among switchers across all landmarks, widening by 2 years (0.725 vs. 0.506; *p* < 0.001). Among patients initiating PD who switched early, differences were not statistically significant at 3 months, 6 months, and 1 year, but 2-year survival was higher among switchers (0.725 vs. 0.571; *p* = 0.022).

**Figure 2:**
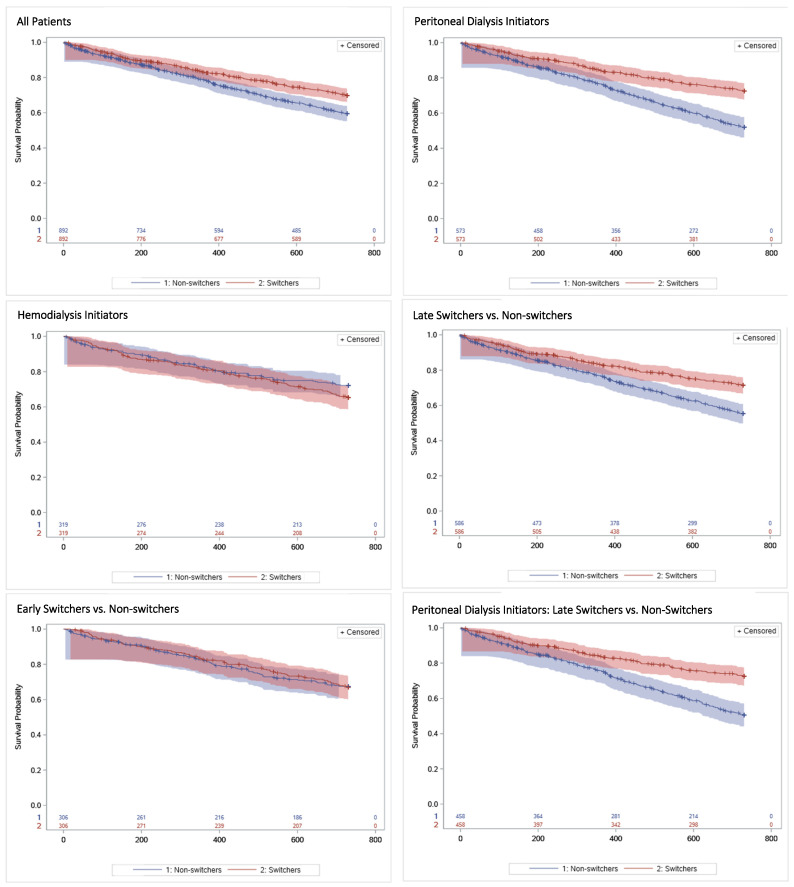

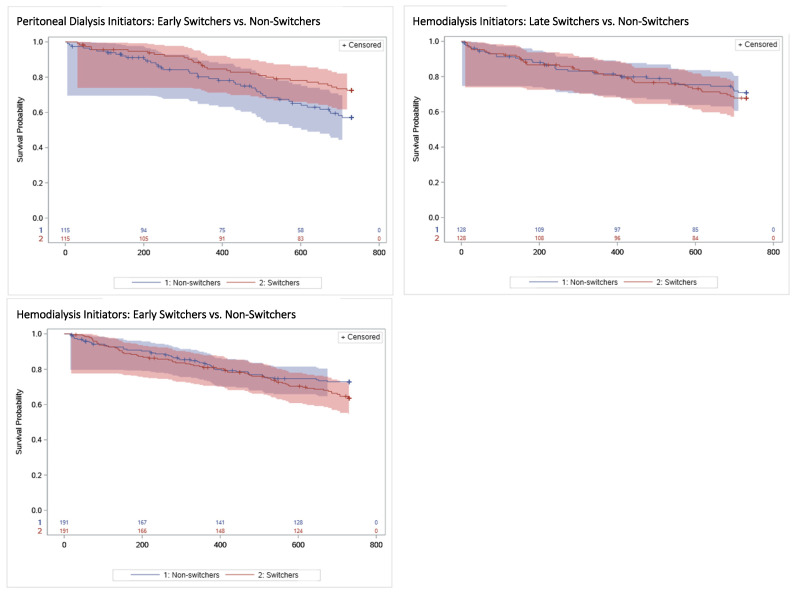
Kaplan–Meier Survival Curves for All-Cause Mortality Comparing Switchers and Non-Switchers From the Start of Follow-Up *. Curves show estimated survival probabilities with 95% confidence intervals. Panel titles indicate the subgroup included in each analysis. “All Patients” includes the entire cohort. “Peritoneal Dialysis Initiators” and “Hemodialysis Initiators” include patients according to their first dialysis modality. “Late Switchers vs. Non-Switchers” and “Early Switchers vs. Non-Switchers” compare switchers according to the timing of the modality switch with their corresponding non-switcher controls. The combined titles, such as “Peritoneal Dialysis Initiators: Late Switchers vs. Non-Switchers”, indicate analyses restricted to patients with the specified initial modality and switch-timing subgroup. Curves were generated at the group level for descriptive purposes. Matched pairs were used only in the regression analysis. Early switch was defined as a switch within 180 days of treatment initiation; late switch was defined as a switch more than 180 days after treatment initiation. * For switchers, follow-up began on the date of the qualifying dialysis modality change. For non-switchers, follow-up began at the dialysis initiation date plus the corresponding time elapsed from the matched switcher’s initiation to treatment change.

Among patients initiating HD, estimates were similar between switchers and non-switchers regardless of whether the switch was late or early (Figure 2 and Supplementary Table S1).

### 3.3. Multivariable Models

As seen in Table 2, overall, compared with non-switchers, switchers had similar adjusted mortality hazards at 3 and 6 months, but lower 1-year and 2-year HRs for mortality (0.723 [95% CI: 0.557–0.938] and 0.652 [0.529–0.803], respectively). Associations differed by subgroup: among patients initiating HD, switchers did not differ from non-switchers across follow-up times, whereas among those initiating PD, switch was associated with lower mortality hazards at 6 months, 1 year, and 2 years (0.600 [0.388–0.928], 0.572 [0.408–0.802], and 0.461 [0.348–0.611], respectively). Timing analyses showed null associations for early switch (≤180 days) but lower mortality hazards among switchers who underwent late switch (>180 days), significantly at 1 and 2 years (0.611 [0.444–0.841] and 0.515 [0.394–0.673], respectively; *p* < 0.01).

In combined modality–timing strata, PD-first late switchers had consistently lower mortality hazards across follow-up (e.g., 2-year HR, 0.421 [0.306–0.579]), whereas HD-first early switchers had higher 2-year mortality hazards (1.614 [0.951–2.740]), with borderline significance (*p* = 0.07).

Results were broadly similar in the sensitivity analyses of censoring at the second switch (Supplementary Table S2) and with a 90-day cut-off for early vs. late switch (Supplementary Table S3).

### 3.4. Causes of Death Findings

Overall, 591 participants died within 2 years of follow-up initiation (258 switchers; 333 non-switchers). In this cohort, switchers were more likely to have initiated HD and were relatively more common in earlier incident-year cohorts (*p* < 0.05 for both) (Supplementary Table S4).

Cause-of-death information was unavailable for 74 decedents; therefore, analyses of primary underlying causes were based on 517 deaths. The distribution of the primary underlying cause of death was similar between switchers and non-switchers. In both groups, “Other” causes comprised the largest share, while heart disease (20.1% in switchers, 21.3% in non-switchers) and renal disease (20.5% in switchers, 18.0% in non-switchers) were also prominent primary causes.

For multiple causes of death, renal disease was the most commonly recorded contributor overall (71.3% in switchers, 63.7% in non-switchers), followed by heart disease (53.7% in switchers, 54.2% in non-switchers); these patterns were largely comparable across groups, with only a borderline difference suggesting renal disease may have been recorded more often among switchers than non-switchers (*p* = 0.07) (Supplementary Table S4).

**Figure 3:**
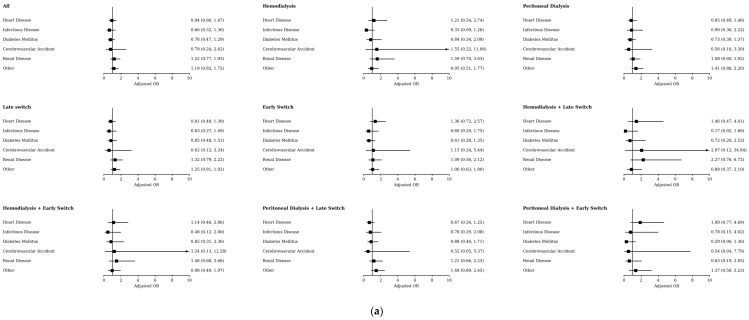

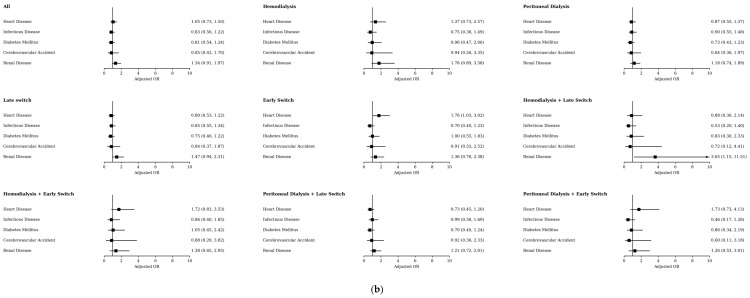
(**a**) Odds of Cause-of-Death Categories Among Decedents Within Two Years: Switchers vs. Non-Switchers (Primary Cause). (**b**) Odds of Cause-of-Death Categories Among Decedents Within Two Years: Switchers vs. Non-Switchers (Multiple Causes). Early switch was defined as a switch within 180 days of treatment initiation; late switch was defined as a switch more than 180 days after treatment initiation. In analyses stratified by initial modality, models were adjusted for age group, sex, population group, peripherality, and incident-year cohort; in all other analyses, initial modality was additionally included as a covariate. Abbreviations: OR, Odds ratio.

In logistic regression analyses of cause-of-death categories among decedents within two years, switch status was not associated with a different distribution of the primary underlying cause of death in either unadjusted or adjusted models, and this pattern remained similar when models were stratified by initial dialysis modality and/or by switch timing (Figure 3a and Supplementary Table S5).

For multiple causes of death, results were likewise broadly comparable overall; however, early switchers had higher adjusted odds of heart disease (OR 1.765, 95% CI 1.032–3.019), and among patients initiating HD, late switchers had higher adjusted odds of renal disease as a contributing cause compared with non-switchers (OR 3.645, 95% CI 1.154–11.511) (Figure 3b and Supplementary Table S5).

## 4. Discussion

This matched cohort study using data from the INRRTR suggests that the association between dialysis modality switch and mortality is not uniform but may vary according to the direction and timing of transition. We found that a late transition from PD to HD was associated with more favorable survival, whereas an early transition from HD to PD was associated with higher long-term mortality, with borderline statistical significance. However, due to methodological limitations, including residual confounding, the observed associations may reflect patient selection and survivor effects rather than a causal effect of the transition itself and therefore should be interpreted primarily as reflecting differences in patient trajectories. In addition, the distribution of primary causes of death, predominantly cardiovascular and renal disease, was broadly similar among switchers and non-switchers.

### 4.1. Comparison to Previous Studies

Prior literature on modality switches and mortality has been mixed, reflecting differences in clinical contexts, policy frameworks, comparison groups, and how transition pathways are defined and analyzed. In line with our findings, evidence from other settings has suggested a survival benefit for PD-to-HD switchers. Van Biesen et al. (2000) (*n* = 417) reported a more favorable prognosis for PD-to-HD switchers (*n* = 32), who achieved a median survival of 95.7 months compared with 38.4 months for patients remaining on PD [28]. Likewise, Lukowsky et al. (2013), in a registry analysis of 23,718 U.S. patients with two-year follow-up, found that a switch in either direction occurring after the first 90 days was associated with improved survival compared with remaining on the initial modality (HR 0.52, 95% CI: 0.41–0.65) [27]. Sangthawan et al. (2025) [14], analyzing a large national registry in Thailand (*n* = 81,572) under a national PD-first policy, reported the highest survival among patients transitioning from PD to HD (defined as a switch lasting ≥60 consecutive days). Using HD-only patients as the reference, PD-to-HD switchers had a 48% lower all-cause mortality risk (95% CI: 0.50–0.55), whereas PD-only patients had a higher risk (HR 1.28, 95% CI: 1.24–1.32), and HD-to-PD switchers had a modestly higher risk (HR 1.12, 95% CI: 1.08–1.16). The authors interpreted these findings as reflecting proactive, flexible transitions to HD at early signs of PD inadequacy [14].

Our observation of a tendency towards increased long-term mortality among patients initiating HD and switching early to PD is in line with the overall direction of the association in several previous investigations. Nessim et al. (2015), in 13,161 Canadian patients, reported substantially higher mortality hazards during the first year on PD among HD-to-PD switchers within the first year of therapy compared with PD-first patients (HR 1.82, 95% CI: 1.52–2.18 for switches ≤90 days; HR 3.11, 95% CI: 2.43–3.98 for 91–180 days) [15]. Nguyen et al. (2019), using Australia and New Zealand Dialysis and Transplant Registry (ANZDATA) data (*n* = 20,882), similarly reported higher mortality hazards among HD-to-PD switchers (after at least three months on HD) compared with patients initiating and remaining on PD (HR 1.335; 95% CI: 1.172–1.520) [11]. A systematic review and meta-analysis by Wang et al. (2020) of eight studies concluded that transfer from HD to PD after ≥3 months on HD was associated with a higher mortality (OR 2.08; 95% CI: 1.09–3.98) compared with PD-first patients [29].

Several studies have reported no statistically significant survival differences across specific switch pathways. For example, the CHOICE study (Jaar et al., 2009; *n* = 262) observed no survival difference among incident PD patients who switched to HD versus those who remained on PD [30]. Pajek et al. (2014) (*n* = 286) likewise reported comparable hazards when restricting to PD-to-HD switchers who survived beyond 60 days post-transition compared with patients staying on PD [13]. In the HD-to-PD direction, Barone et al. (2014) [31] and Najafi et al. (2012) [32] reported broadly equivalent patient and technique survival among PD-first patients and those transferred from HD, suggesting that prior HD exposure did not necessarily compromise subsequent PD outcomes in those cohorts.

Other studies report patterns opposite to ours. For PD-to-HD, Tsai et al. (2022), in a Taiwanese cohort (*n* = 5500), found higher mortality among switchers (after ≥3 months on PD) compared with patients remaining on HD (HR 1.36; 95% CI: 1.21–1.53), with a pronounced early excess risk during the first five months post-transition (HR 2.61; 95% CI: 2.04–3.35) [26]. A multinational analysis of >114,000 incident PD patients (Nadeau-Fredette et al., 2022) likewise reported that mortality was highest in the first 30 days after transfer from PD to HD across registries, compared with subsequent follow-up periods on HD, framing the transition as a period of peak vulnerability [12]. Regarding HD-to-PD, while we identified early switchers as a high-risk group, Lukowsky et al. (2013) reported that a switch after the initial 90 days was associated with improved survival compared with remaining on the initial modality [27].

Taken together, the variability in reported mortality associations likely reflects differences in (i) healthcare policy and care delivery (e.g., integrated PD-first systems versus mixed or HD-dominant systems), (ii) whether transitions are typically planned or precipitated by acute instability (e.g., access failure, refractory peritonitis), and (iii) methodological choices (e.g., landmark definitions, limited availability of granular clinical data, censoring strategies, choice of comparison group, and analytic approaches). These considerations support the interpretation that the association between modality switch and mortality may be shaped not only by modality direction but also by the timing and clinical context in which the transition occurs.

### 4.2. Possible Explanations

Several physiological, clinical, and methodological mechanisms may plausibly explain the trajectory-dependent survival/mortality patterns observed. The lower mortality observed among late PD-to-HD transitions may relate, in part, to better preservation of residual renal function (RRF) during the PD phase. Preserved RRF could serve as a physiological buffer supporting volume control, solute clearance, and metabolic stability, potentially conferring downstream benefits even after transfer to HD [14]. The absence of early survival differences is compatible with a balance between longer-term physiological advantages and short-term hazards concentrated around the transition interval [13].

This transition period may represent a window of increased vulnerability, particularly when transfers are unplanned and involve “crash” starts, reliance on central venous catheters (CVCs) rather than mature access, or acute complications such as refractory peritonitis [7,12]. Timing may also capture different clinical phenotypes: late transitions may more often reflect a proactive or elective switch within an integrated care pathway, whereas early transitions may be more reactive markers of instability or technique failure [14,33].

Beyond biological factors, patient-level behavioral and psychosocial pathways could contribute. Patients established on home-based PD may develop greater self-efficacy and self-monitoring practices, which could plausibly translate into better adherence and engagement after transitioning to facility-based HD [34]. At the same time, these explanations should be viewed cautiously, as survivor bias or a healthy-user effect may also contribute to the more favorable outcomes observed among patients who remained on PD long enough to undergo a late transition [14].

In contrast, the higher mortality hazard observed among early HD-to-PD switchers, albeit with borderline significance, may reflect rapid loss of RRF following hemodynamic stressors early in HD, inflammatory sequelae associated with initial CVC exposure, or subclinical HD-related cardiac injury that could confer longer-term vulnerability even after transfer to PD [15]. Early switch from HD may also be driven by factors that are prognostically adverse but incompletely measured in registry data, including clinical instability, vascular access failure, or limited predialysis preparation, consistent with confounding by indication [15].

### 4.3. Causes of Death Patterns

Although switch status was associated with differences in mortality, the distribution of primary underlying causes of death, mainly cardiovascular and renal disease, was largely consistent across switchers and non-switchers. However, when considering multiple contributing factors, early switchers had 1.7-fold higher adjusted odds of heart disease as a contributory cause, and late switchers from HD had more than threefold higher adjusted odds of renal disease as a contributory cause compared with non-switchers. Notably, these analyses describe cause patterns among decedents and should not be interpreted as cause-specific mortality risks in the full cohort.

The stability in primary causes of death is consistent with prior reports, such as from Zhang et al. (2013), who found no major differences in causes of death between PD-only patients and those transferred from HD, with cardiovascular disease being the main cause in both groups (about 57%) [3]. Similar findings were reported by Pajek et al. (2014) [13]. Conversely, other analyses highlight time-dependent shifts around transitions. Nadeau-Fredette et al. (2022) reported that while cardiovascular disease remains a leading cause overall, infection-related deaths may peak early after PD-to-HD transitions, with cardiovascular deaths becoming more dominant thereafter [12]. Chen et al. (2018) likewise noted that infectious and social causes of technique failure can be linked to higher premature mortality within the first two years after transfer [35].

In the context of our findings, two interpretations are plausible. First, the lack of a major shift in the primary underlying causes of death suggests that the observed mortality differences likely reflect a shift in the timing of deaths from the same leading causes, rather than a change in the causes themselves or the emergence of new fatal mechanisms. Second, the higher burden of renal disease documented as a contributing cause among HD-first late switchers may reflect greater end-of-life clinical complexity and a more prolonged course of renal-related complications, potentially capturing renal-related morbidity that also contributed to, or prompted, the decision to switch modalities. In addition, early modality switchers may represent a more clinically unstable subgroup with a higher underlying cardiovascular burden. Rapid transitions may be driven by acute complications linked with heart disease, which may increase the likelihood of cardiovascular conditions contributing to death.

### 4.4. Strengths and Limitations

A key strength of this study is the use of a large, contemporary, nationally representative source of data, with complete RRT trajectories over a 13-year period (2010–2022). The use of a 30-day rule to define modality episodes helps ensure that switches represent sustained clinical transitions rather than transient procedural changes. The matched design also reduces immortal time bias by pairing each switcher with a non-switcher who initiated therapy on the same modality and survived at least as long as the time to the switcher’s transition.

Importantly, the analysis stratified transitions by both direction (PD-to-HD vs. HD-to-PD) and timing (early vs. late), allowing identification of clinically distinct pathways. Finally, linkage with national mortality files from the CBS enabled examination of both primary and multiple contributing causes of death, extending the interpretation of the findings.

Several limitations warrant consideration. As a retrospective registry-based study, the dataset lacks granular clinical information on baseline RRF and dialysis adequacy measures, e.g., Kt/V. In addition, the registry does not include detailed clinical indications for switch (e.g., refractory peritonitis, patient preference, or planned integrated care), which may be more prognostic than the modality itself. Importantly, we also lacked information on arteriovenous access-related complications, including dialysis access steal syndrome, vascular access failure, and cardiac complications related to arteriovenous access, such as high-output heart failure or worsening cardiac burden. These complications may influence both the decision to switch modality and subsequent mortality risk.

We attempted to mitigate this limitation by using transition timing (early vs. late) as a proxy for clinical context; late PD-to-HD transitions may reflect proactive switches, whereas early transitions may more often reflect reactive instability. Nonetheless, unmeasured confounding and selection biases remain possible, including survivor bias/healthy user effects among patients who sustain PD long enough to switch late and “sick stopper” dynamics whereby patients too unstable to switch remain categorized as non-switchers. Conversely, early HD-to-PD switchers may be enriched for baseline frailty, vascular access failure, or acute comorbidity, which could inflate hazard estimates independent of the transition.

In addition, Kaplan–Meier curves were used as descriptive unadjusted group-level displays and did not preserve the matched-pair structure. The matched-pair structure was accounted for in the conditional Cox regression models only. Moreover, although the overall 2-year mortality analysis was adequately powered, some stratified and cause-of-death analyses included relatively small numbers of events; therefore, these subgroup estimates should be interpreted cautiously. Finally, alternative analytic approaches, including time-dependent Cox models or marginal structural models, could address different questions by modeling modality status over the full dialysis course. However, these approaches require more detailed longitudinal information on time-varying variables, which is not available in the registry.

Accordingly, the present findings should be interpreted as associations and a comparison of post-transition trajectories rather than causal effects. While the trajectory-dependent patterns observed here may help inform clinical surveillance and transition planning, they should not be taken to imply that switching itself is beneficial or harmful irrespective of patient context. Further studies with richer clinical detail are needed to clarify the extent to which these findings reflect transition-related factors, baseline clinical status, and residual confounding.

## 5. Conclusions

In this nationally matched cohort, mortality patterns after dialysis modality switch differed by transition direction and timing. Lower long-term mortality among late PD-to-HD switchers and a tendency toward higher mortality among early HD-to-PD switchers suggest that modality transitions identify clinically distinct patient trajectories. Given the observational design and the potential for confounding by indication, survivor effects, and unmeasured clinical factors, these findings should not be interpreted as causal effects of switching itself. Further studies incorporating detailed clinical indications, residual renal function, dialysis adequacy, vascular access, and transition planning are needed.

## Figures and Tables

**Table 1:** Characteristics of Study Participants, 2010–2022 *.

Variable	*N*	All (*N* = 1784)	Switchers (*N* = 892)	Non-Switchers (*N* = 892)	*p*-Value
**Age, Median Years (Q1, Q3)**	1784	65.0 (55.6, 73.2)	64.9 (55.7, 73.4)	65.2 (55.5, 72.8)	0.29
**Age Group, *n* (%)**	1784				1.00
18–44		201 (11.3)	100 (11.2)	101 (11.3)	
45–64		688 (38.6)	350 (39.2)	338 (37.9)	
65–74		535 (30.0)	263 (29.5)	272 (30.5)	
75–84		320 (17.9)	160 (17.9)	160 (17.9)	
85+		40 (2.2)	19 (2.1)	21 (2.4)	
**Sex, *n* (%)**	1784				0.12
Male		1179 (66.1)	605 (67.8)	574 (64.4)	
Female		605 (33.9)	287 (32.2)	318 (35.6)	
**Population Group, *n* (%)**	1784				0.72
Jews and Others ^a^		1392 (78.0)	693 (77.7)	699 (78.4)	
Arabs		392 (22.0)	199 (22.3)	193 (21.6)	
**Socioeconomic Status, *n* (%)**	1737				0.58
Low		356 (20.5)	182 (20.8)	174 (20.2)	
Medium		758 (43.6)	372 (42.5)	386 (44.8)	
High		623 (35.9)	321 (36.7)	302 (35.0)	
**Peripherality, *n* (%)**	1776				0.36
Peripheral		389 (21.9)	200 (22.4)	189 (21.3)	
Intermediate		279 (15.7)	151 (17.0)	128 (14.5)	
Central		1108 (62.4)	539 (60.6)	569 (64.2)	
**Orthodoxy level, *n* (%)**	1784				**0.047**
Low		1707 (95.7)	862 (96.6)	845 (94.7)	
High		77 (4.3)	30 (3.4)	47 (5.3)	
**First Modality, *n* (%)**	1784				1.00
Peritoneal Dialysis		1146 (64.2)	573 (64.2)	573 (64.2)	
Hemodialysis		638 (35.8)	319 (35.8)	319 (35.8)	
**Incident-Year Cohort, *n* (%)**	1784				**<0.001**
2010–2013		495 (27.7)	280 (31.4)	215 (24.1)	
2014–2017		649 (36.4)	333 (37.3)	316 (35.4)	
2018–2022		640 (35.9)	279 (31.3)	361 (40.5)	
**Facility Type- All Participants, *n* (%) ^c^**	1784				**<0.001**
Hospital		1610 (90.3)	827 (92.7)	783 (87.8)	
Community		174 (9.7)	65 (7.3)	109 (12.2)	
**Facility Type- Hemodialysis Initiators Only, *n* (%)** ^c^	638				**<0.001**
Hospital		464 (72.7)	254 (79.6)	210 (65.8)	
Community		174 (27.3)	65 (20.4)	109 (34.2)	
**Primary Renal Disease, *n* (%)**	1784				**0.039**
Glomerulonephritis		164 (9.2)	92 (10.3)	72 (8.1)	
Diabetes Mellitus		802 (45.0)	420 (47.1)	382 (42.8)	
Hypertension/Renal Vascular Disease		209 (11.7)	108 (12.1)	101 (11.3)	
Other		303 (17.0)	143 (16.0)	160 (17.9)	
Unknown/Missing		306 (17.1)	129 (14.5)	177 (19.8)	
**Time Until Switching, Median Days (Q1, Q3)**	892	NA	324 (120, 712)	NA	**NA**
**Time to Modality Switch ^b^**	1784				1.00
Early Switch (≤180 Days)		612 (34.3)	306 (34.3)	306 (34.3)	
Late Switch (>180 Days)		1172 (65.7)	586 (65.7)	586 (65.7)	

* Exposure groups were matched based on 1:1 matching. Each switcher was matched to a non-switcher control by: (A) age (±5 years); (B) initial treatment modality (HD or PD); (C) to ensure comparable follow-up opportunities, the matched control was required to survive at least the same duration as the time elapsed from the switcher’s treatment initiation until the date of treatment change. ^a^ The “Others” category encompasses individuals who do not identify as Jewish or Arab. This includes non-Arab Christians, members of other religions, and individuals without a religious classification. ^b^ For non-switchers, category assignment was determined according to the matched switcher. ^c^ Facility type is presented for the overall cohort and separately for hemodialysis initiators, as in Israel, peritoneal dialysis occurs exclusively in hospital-based settings. *p*-values with bold font indicate statistical significance (*p* < 0.05). NA-Not available.

**Table 2:** Adjusted Conditional Cox Proportional Hazards Regression Results for the Association Between Dialysis Modality Switching and Mortality, 2010–2022 (Without Censoring at the Second Switch) *.

		3-Month Mortality		6-Month Mortality		1-Year Mortality		2-Year Mortality	
Outcome: Mortality	*N* ** (Missing)	Adjusted Hazard Ratio (95% Confidence Interval)	*p*-Value	Adjusted Hazard Ratio (95% Confidence Interval)	*p*-Value	Adjusted Hazard Ratio (95% Confidence Interval)	*p*-Value	Adjusted Hazard Ratio (95% Confidence Interval)	*p*-Value
**All ^a^**	1776 (8)		0.11		0.18		**0.015**		**<0.001**
Non-Switchers		Reference		Reference		Reference		Reference	
Switchers		0.692 (0.439, 1.092)		0.796 (0.568, 1.114)		0.723 (0.557, 0.938)		0.652 (0.529, 0.803)	
**First Modality: Peritoneal Dialysis ^b^**	1144 (2)		0.11		**0.022**		**0.001**		**<0.001**
Non-Switchers		Reference		Reference		Reference		Reference	
Switchers		0.607 (0.327, 1.125)		0.600 (0.388, 0.928)		0.572 (0.408, 0.802)		0.461 (0.348, 0.611)	
**First Modality: Hemodialysis ^a^**	632 (6)		0.88		0.31		0.55		0.36
Non-Switchers		Reference		Reference		Reference		Reference	
Switchers		1.065 (0.457, 2.478)		1.383 (0.738, 2.593)		1.147 (0.729, 1.806)		1.181 (0.826, 1.690)	
**Late Switch ^a^**	1171 (1)		0.08		0.06		**0.003**		**<0.001**
Non-Switchers		Reference		Reference		Reference		Reference	
Switchers		0.605 (0.346, 1.055)		0.684 (0.461, 1.015)		0.611 (0.444, 0.841)		0.515 (0.394, 0.673)	
**Early Switch ^a^**	605 (7)		0.48		0.21		0.61		0.96
Non-Switchers		Reference		Reference		Reference		Reference	
Switchers		1.579 (0.441, 5.654)		1.711 (0.737, 3.975)		1.145 (0.684, 1.917)		1.010 (0.700, 1.458)	
**Peritoneal Dialysis as First Modality + Late Switch ^b^**	915 (1)		**0.032**		**0.020**		**<0.001**		**<0.001**
Non-Switchers		Reference		Reference		Reference		Reference	
Switchers		0.430 (0.199, 0.930)		0.576 (0.362, 0.917)		0.528 (0.364, 0.766)		0.421 (0.306, 0.579)	
**Peritoneal Dialysis as First Modality + Early Switch ^b^**	229 (1)		NA		NA		0.69		0.15
Non-Switchers		Reference		Reference		Reference		Reference	
Switchers		NA		NA		0.791 (0.255, 2.461)		0.572 (0.266, 1.230)	
**Hemodialysis as First Modality + Late Switch ^a^**	256 (0)		0.83		0.82		0.75		0.69
Non-Switchers		Reference		Reference		Reference		Reference	
Switchers		1.349 (0.088, 20.723)		1.164 (0.321, 4.224)		1.138 (0.512, 2.528)		0.887 (0.489, 1.609)	
**Hemodialysis as First Modality + Early Switch ^a^**	376 (6)		0.65		0.16		0.22		0.07
Non-Switchers		Reference		Reference		Reference		Reference	
Switchers		1.711 (0.174, 16.821)		2.067 (0.746, 5.729)		1.559 (0.766, 3.170)		1.614 (0.951, 2.740)	

Early switch was defined as switching within 180 days of treatment initiation; late switch was defined as switching more than 180 days after treatment initiation. * Exposure groups were matched based on 1:1 matching. Each switcher was matched to a non-switcher control by: (A) age (±5 years); (B) initial treatment modality (hemodialysis or peritoneal dialysis); (C) to ensure comparable follow-up opportunities, the matched control was required to survive at least the same duration as the time elapsed from the switcher’s treatment initiation until the date of treatment change. ** *N* represents the number of observations included in each model. ^a^ Analyses were adjusted for sex, population group, peripherality, orthodoxy level, incident-year cohort, facility type, and primary renal disease. ^b^ Analyses were adjusted for sex, population group, peripherality, orthodoxy level, incident-year cohort, and primary renal disease. *p*-values with bold font indicate statistical significance (*p* < 0.05). NA—Not available. Given the limited sample size, multivariable adjustment could not be performed.

## Data Availability

The data presented in this study are not publicly available due to privacy and regulatory restrictions related to individual-level national registry data. Data may be made available by the corresponding author upon reasonable request and subject to approval by the relevant regulatory authorities.

## Supplementary Materials

The following supporting information can be downloaded at: https://www.mdpi.com/article/10.3390/jcm15103948/s1, Supplementary Table S1: All-Cause Survival Probabilities at 3 Months, 6 Months, 1 Year, and 2 Years According to Dialysis Modality Switching Status (Kaplan–Meier Estimates); Supplementary Table S2: Adjusted Conditional Cox Proportional Hazards Regression Results for the Association Between Dialysis Modality Switching and Mortality, 2010–2022 (With Censoring at the Second Switch); Supplementary Table S3: Adjusted Conditional Cox Proportional Hazards Regression Results for the Association Between Dialysis Modality Switching and Mortality, 2010–2022, by Switch Timing (Within or Over 90 Days); Supplementary Table S4: Characteristics and Causes of Death of Participants Who Died Within 2 Years After Follow-up Initiation, 2010–2022; Supplementary Table S5: Unadjusted and Adjusted Odds Ratios for Cause-Specific Death Within Two Years of Follow-Up Among Dialysis Modality Switchers Compared With Non-Switchers.

## Abbreviations

ANZDATA	Australia and New Zealand Dialysis and Transplant Registry
CBS	Central Bureau of Statistics
CI	Confidence interval
CVC	Central venous catheter
CVA	Cerebrovascular disease
EDTA	European Dialysis and Transplant Association
ERA-EDTA	European Renal Association–European Dialysis and Transplant Association
ESRD	End-stage renal disease
HD	Hemodialysis
HR	Hazard ratio
ICD	International Classification of Diseases
INRRTR	Israeli National Renal Replacement Therapy Registry
NA	Not available
OR	Odds ratio
PD	Peritoneal dialysis
PLI	Points Location Intelligence
Q1	First quartile
Q3	Third quartile
RRF	Residual renal function
RRT	Renal replacement therapy
SES	Socioeconomic status
SGAs	Statistical Geographic Areas
