# Supplementary material for: Outcomes of Dialysis Modality Switch: A Matched Cohort Analysis from a National Renal Replacement Therapy Registry, 2010–2022
Source: J Clin Med. 2026 May 20;15(10):3948. doi: 10.3390/jcm15103948 (PMC13207222; doi:10.3390/jcm15103948)
Supplement: Supplementary file 1 [file jcm-15-03948-s001.zip › Supp. Table S1 Switch 19.4.pdf]

Supplementary Table S1. All-Cause Survival Probabilities at 3 Months, 6 Months, 1 Year, and 2 Years According to Dialysis Modality Switching Status (Kaplan–Meier Estimates)*

	**3-Month Survival**		**6-Month Survival**		**1-Year Survival**		**2-Year Survival**	
	**Survival Probability (95% Confidence Interval)**	**P-value**	**Survival Probability (95% Confidence Interval)**	**P-value**	**Survival Probability (95% Confidence Interval)**	**P-value**	**Survival Probability (95% Confidence Interval)**	**P-value**
All	0.939 (0.927, 0.949)		0.891 (0.846, 0.905)		0.806 (0.787, 0.824)		0.648 (0.624, 0.671)	
**All, by Switching Status**		0.06		0.24		**0.023**		**<.001**
Switchers	0.949 (0.933, 0.962)		0.899 (0.877, 0.917)		0.827 (0.800, 0.851)		0.698 (0.666, 0.728)	
Non-Switchers	0.929 (0.909, 0.944)		0.883 (0.860, 0.903)		0.785 (0.756, 0.811)		0.596 (0.561, 0.629)	
**First Modality: Peritoneal Dialysis**		**0.027**		**0.026**		**0.001**		**<.001**
Switchers	0.958 (0.938, 0.972)		0.912 (0.885, 0.932)		0.837 (0.804, 0.866)		0.725 (0.684, 0.761)	
Non-Switchers	0.928 (0.903, 0.946)		0.871 (0.840, 0.896)		0.762 (0.723, 0.796)		0.519 (0.473, 0.562)	
**First Modality: Hemodialysis**		0.81		0.32		0.63		0.11

Switchers	0.934 (0.901, 0.957)		0.877 (0.836, 0.909)		0.809 (0.761, 0.849)		0.652 (0.595, 0.703)	
Non-Switchers	0.930 (0.896, 0.953)		0.904 (0.866, 0.932)		0.825 (0.778, 0.863)		0.722 (0.667, 0.769)	
**Late Switch**		**0.027**		0.15		**0.008**		**<.001**
Switchers	0.952 (0.931, 0.967)		0.895 (0.867, 0.917)		0.829 (0.796, 0.858)		0.715 (0.675, 0.751)	
Non-Switchers	0.921 (0.895, 0.940)		0.869 (0.838, 0.894)		0.766 (0.728, 0.799)		0.553 (0.509, 0.595)	
**Early Switch**		0.94		0.96		0.90		0.97
Switchers	0.944 (0.911, 0.965)		0.907 (0.869, 0.935)		0.823 (0.775, 0.862)		0.669 (0.611, 0.719)	
Non-Switchers	0.944 (0.911, 0.965)		0.910 (0.871, 0.937)		0.821 (0.772, 0.861)		0.675 (0.616, 0.727)	
**Peritoneal Dialysis + Late Switch**		**0.022**		**0.046**		**0.002**		**<.001**
Switchers	0.958 (0.936, 0.973)		0.903 (0.871, 0.927)		0.835 (0.797, 0.867)		0.725 (0.680, 0.765)	
Non-Switchers	0.923 (0.894, 0.944)		0.861 (0.825, 0.890)		0.752 (0.708, 0.790)		0.506 (0.455, 0.555)	
**Peritoneal Dialysis + Early Switch**		0.75		0.30		0.33		**0.022**
Switchers	0.956 (0.898, 0.982)		0.947 (0.886, 0.976)		0.846 (0.764, 0.902)		0.725 (0.630, 0.799)	
Non-Switchers	0.948 (0.887, 0.976)		0.911 (0.841, 0.951)		0.802 (0.712, 0.866)		0.571 (0.465, 0.663)	
**Hemodialysis + Late Switch**		0.63		0.50		0.92		0.65
Switchers	0.930 (0.869, 0.963)		0.866 (0.794, 0.915)		0.808 (0.727, 0.867)		0.678 (0.586, 0.754)	

Non-Switchers	0.913 (0.848, 0.951)		0.897 (0.829, 0.939)		0.815 (0.735, 0.873)		0.709 (0.619, 0.782)	
**Hemodialysis + Early Switch**		0.90		0.47		0.59		0.10
Switchers	0.937 (0.892, 0.964)		0.884 (0.830, 0.922)		0.810 (0.746, 0.859)		0.635 (0.560, 0.700)	
Non-Switchers	0.942 (0.897, 0.967)		0.909 (0.858, 0.943)		0.832 (0.770, 0.879)		0.729 (0.658, 0.788)	

Early switch was defined as switching within 180 days of treatment initiation; late switch was defined as switching more than 180 days after treatment initiation.

*For switchers, follow-up began at the date of the qualifying dialysis modality change. For non-switchers, follow-up began at the dialysis initiation date plus the corresponding time elapsed from the matched switcher's initiation to treatment change.
