# Supplementary material for: Outcomes of Dialysis Modality Switch: A Matched Cohort Analysis from a National Renal Replacement Therapy Registry, 2010–2022
Source: J Clin Med. 2026 May 20;15(10):3948. doi: 10.3390/jcm15103948 (PMC13207222; doi:10.3390/jcm15103948)
Supplement: Supplementary file 1 [file jcm-15-03948-s001.zip › Supp. Table S2 Switch 19.4.pdf]

Supplementary Table S2. Adjusted Conditional Cox Proportional Hazards Regression Results for the Association Between Dialysis Modality Switching and Mortality, 2010–2022 (With Censoring at the Second Switch)*

		**3-Month Mortality**		**6-Month Mortality**		**1-Year Mortality**		**2-Year Mortality**	
**Outcome: Mortality**	**N** (Missing)**	**Adjusted Hazard Ratio (95% Confidence Interval)**	**P-value**	**Adjusted Hazard Ratio (95% Confidence Interval)**	**P-value**	**Adjusted Hazard Ratio (95% Confidence Interval)**	**P-value**	**Adjusted Hazard Ratio (95% Confidence Interval)**	**P-value**
**All[a]**	1776 (8)		0.09		0.15		**0.022**		**<.001**
Non-Switchers		Reference		Reference		Reference		Reference	
Switchers		0.666 (0.417, 1.065)		0.776 (0.549, 1.098)		0.731 (0.558, 0.956)		0.647 (0.518, 0.808)	
**First Modality: Peritoneal Dialysis[b]**	1144 (2)		0.11		**0.018**		**0.002**		**<.001**
Non-Switchers		Reference		Reference		Reference		Reference	
Switchers		0.607 (0.327, 1.125)		0.583 (0.373, 0.912)		0.585 (0.414, 0.826)		0.476 (0.356, 0.636)	

**First Modality: Hemodialysis[a]**	632 (6)		0.63		0.49		0.70		0.46
Non-Switchers		Reference		Reference		Reference		Reference	
Switchers		0.788 (0.300, 2.072)		1.269 (0.648, 2.486)		1.101 (0.679, 1.786)		1.167 (0.777, 1.752)	
**Early Switch[a]**	605 (7)		0.84		0.26		0.73		0.90
Non-Switchers		Reference		Reference		Reference		Reference	
Switchers		0.870 (0.216, 3.498)		1.633 (0.702, 3.799)		1.098 (0.641, 1.883)		0.975 (0.657, 1.447)	
**Late Switch[a]**	1171 (1)		0.07		**0.048**		**0.006**		**<.001**
Non-Switchers		Reference		Reference		Reference		Reference	
Switchers		0.586 (0.327, 1.050)		0.660 (0.437, 0.997)		0.626 (0.449, 0.871)		0.518 (0.390, 0.688)	
**Peritoneal Dialysis as First Modality + Late Switch[b]**	915 (1)		**0.032**		**0.016**		**0.001**		**<.001**
Non-Switchers		Reference		Reference		Reference		Reference	
Switchers		0.430 (0.199, 0.930)		0.554 (0.343, 0.895)		0.536 (0.366, 0.785)		0.438 (0.315, 0.607)	

**Peritoneal Dialysis as First Modality + Early Switch[b]**	229 (1)		NA		NA		0.89		0.21
Non-Switchers		Reference		Reference		Reference		Reference	
Switchers		NA		NA		0.917 (0.283, 2.972)		0.612 (0.285, 1.314)	
**Hemodialysis as First Modality + Late Switch[a]**	256 (0)		NA		0.48		0.38		0.41
Non-Switchers		Reference		Reference		Reference		Reference	
Switchers		NA		3.438 (0.108, 109.618)		1.724 (0.507, 5.860)		0.695 (0.293, 1.646)	
**Hemodialysis as First Modality + Early Switch[a]**	376 (6)		NA		0.24		0.43		0.14
Non-Switchers		Reference		Reference		Reference		Reference	
Switchers		NA		1.867 (0.666, 5.230)		1.348 (0.641, 2.831)		1.562 (0.863, 2.824)	

Early switch was defined as switching within 180 days of treatment initiation; late switch was defined as switching more than 180 days after treatment initiation.

*Exposure groups were matched based on 1:1 matching. Each switcher was matched to a non-switcher control by: (A) age (±5 years); (B) initial treatment modality (hemodialysis or peritoneal dialysis); (C) to ensure comparable follow-up opportunities, the matched control was required to survive at least the same duration as the time elapsed from the switcher's treatment initiation until the date of treatment change.

**N represents the number of observations included in each model.

[a]Analyses were adjusted for sex, population group, peripherality, orthodoxy level, incident-year cohort, facility type, and primary renal disease.

[b]Analyses were adjusted for sex, population group, peripherality, orthodoxy level, incident-year cohort, and primary renal disease.

P-values with bold font indicate statistical significance ($p<0.05$).

NA – Not available; Given the limited sample size, multivariable adjustment could not be performed.
