# Supplementary material for: Outcomes of Dialysis Modality Switch: A Matched Cohort Analysis from a National Renal Replacement Therapy Registry, 2010–2022
Source: J Clin Med. 2026 May 20;15(10):3948. doi: 10.3390/jcm15103948 (PMC13207222; doi:10.3390/jcm15103948)
Supplement: Supplementary file 1 [file jcm-15-03948-s001.zip › Supp. Table S3 Switch 19.4.pdf]

Supplementary Table S3. Adjusted Conditional Cox Proportional Hazards Regression Results for the Association Between Dialysis Modality Switching and Mortality, 2010–2022, by Switch Timing (Within or Over 90 Days)*

		**3-Month Mortality**		**6-Month Mortality**		**1-Year Mortality**		**2-Year Mortality**	
**Outcome: Mortality**	**N** (Missing)**	**Adjusted Hazard Ratio (95% Confidence Interval)**	**P-value**	**Adjusted Hazard Ratio (95% Confidence Interval)**	**P-value**	**Adjusted Hazard Ratio (95% Confidence Interval)**	**P-value**	**Adjusted Hazard Ratio (95% Confidence Interval)**	**P-value**
**Late Switch[a]**	1435 (5)		0.07		**0.039**		**0.002**		**<.001**
Non-Switchers		Reference		Reference		Reference		Reference	
Switchers		0.620 (0.368, 1.042)		0.669 (0.457, 0.980)		0.615 (0.453, 0.834)		0.561 (0.439, 0.716)	
**Early Switch[a]**	341 (3)		0.70		0.14		0.33		0.56
Non-Switchers		Reference		Reference		Reference		Reference	
Switchers		1.772 (0.093, 33.718)		2.262 (0.765, 6.687)		1.378 (0.725, 2.618)		1.152 (0.716, 1.852)	

**Peritoneal Dialysis as First Modality + Late Switch[b]**	1040 (2)		0.08		**0.019**		**<.001**		**<.001**
Non-Switchers		Reference		Reference		Reference		Reference	
Switchers		0.556 (0.291, 1.061)		0.585 (0.374, 0.914)		0.529 (0.369, 0.759)		0.431 (0.319, 0.582)	
**Peritoneal Dialysis as First Modality + Early Switch[b]**	104 (0)		NA		NA		NA		0.39
Non-Switchers		Reference		Reference		Reference		Reference	
Switchers		NA		NA		NA		0.533 (0.129, 2.209)	
**Hemodialysis as First Modality + Late Switch[a]**	395 (3)		0.31		0.64		0.85		0.99
Non-Switchers		Reference		Reference		Reference		Reference	
Switchers		0.332 (0.040, 2.726)		0.778 (0.274, 2.210)		0.934 (0.454, 1.921)		0.997 (0.558, 1.780)	

**Hemodialysis as First Modality + Early Switch[a]**	237 (3)		NA		0.10		0.34		0.26
Non-Switchers		Reference		Reference		Reference		Reference	
Switchers		NA		4.796 (0.734, 31.340)		1.493 (0.658, 3.386)		1.458 (0.753, 2.821)	

Early switch was defined as switching within 90 days of treatment initiation; late switch was defined as switching more than 90 days after treatment initiation.

*Exposure groups were matched based on 1:1 matching. Each switcher was matched to a non-switcher control by: (A) age (±5 years); (B) initial treatment modality (hemodialysis or peritoneal dialysis); (C) to ensure comparable follow-up opportunities, the matched control was required to survive at least the same duration as the time elapsed from the switcher's treatment initiation until the date of treatment change.

**N represents the number of observations included in each model.

[a]Analyses were adjusted for sex, population group, peripherality, orthodoxy level, incident-year cohort, facility type, and primary renal disease.

[b]Analyses were adjusted for sex, population group, peripherality, orthodoxy level, incident-year cohort, and primary renal disease.

P-values with bold font indicate statistical significance ($p<0.05$).

NA – Not available; Given the limited sample size, multivariable adjustment could not be performed.
