# Supplementary material for: Outcomes of Dialysis Modality Switch: A Matched Cohort Analysis from a National Renal Replacement Therapy Registry, 2010–2022
Source: J Clin Med. 2026 May 20;15(10):3948. doi: 10.3390/jcm15103948 (PMC13207222; doi:10.3390/jcm15103948)
Supplement: Supplementary file 1 [file jcm-15-03948-s001.zip › Supp. Table S4 Switch 19.4.pdf]

Supplementary Table S4. Characteristics and Causes of Death of Participants Who Died Within 2 Years After Follow-up Initiation*, 2010–2022

Variable	N	All (N=591)	Switchers (N=258)	Non-Switchers (N=333)	P-Value
**Age, Median Years (Q1, Q3)***	591	69.8 (63.0, 77.2)	69.7 (62.3, 76.7)	69.8 (63.4, 77.6)	0.47
**Age Group, n (%)**	591				0.54
18-44		19 (3.2)	7 (2.7)	12 (3.6)	
45-64		175 (29.6)	85 (33.0)	90 (27.0)	
65-74		213 (36.0)	89 (34.5)	124 (37.2)	
75-84		156 (26.4)	67 (26.0)	89 (26.7)	
85+		28 (4.7)	10 (3.9)	18 (5.4)	
**Sex, n (%)**	591				0.65
Male		409 (69.2)	176 (68.2)	233 (70.0)	
Female		182 (30.8)	82 (31.8)	100 (30.0)	
**Population Group, n (%)**	591				0.26
Jews and Others[a]		466 (78.9)	209 (81.0)	257 (77.2)	
Arabs		125 (21.2)	49 (19.0)	76 (22.8)	
**Socioeconomic Status, n (%)**	577				0.54
Low		106 (18.4)	42 (16.6)	64 (19.8)	
Medium		257 (44.5)	118 (46.6)	139 (42.9)	
High		214 (37.1)	93 (36.8)	121 (37.4)	
**Peripherality, n (%)**	590				0.47
Peripheral		135 (22.9)	65 (25.3)	70 (21.0)	
Intermediate		89 (15.1)	38 (14.8)	51 (15.3)	
Central		366 (62.0)	154 (59.9)	212 (63.7)	
**Orthodoxy level, n (%)**	591				0.43

Low		566 (95.8)	249 (96.5)	317 (95.2)	
High		25 (4.2)	9 (3.5)	16 (4.8)	
**First Modality, n (%)**	591				**<.001**
Peritoneal Dialysis		397 (67.2)	150 (58.1)	247 (74.2)	
Hemodialysis		194 (32.8)	108 (41.9)	86 (25.8)	
**Incident-Year Cohort, n (%)**	591				**0.008**
2010-2013		168 (28.4)	85 (33.0)	83 (24.9)	
2014-2017		204 (34.5)	95 (36.8)	109 (32.7)	
2018-2022		219 (37.1)	78 (30.2)	141 (42.3)	
**Facility Type, n (%)**	591				0.69
Hospital		548 (92.7)	238 (92.3)	310 (93.1)	
Community		43 (7.3)	20 (7.8)	23 (6.9)	
**Primary Renal Disease, n (%)**	591				0.72
Glomerulonephritis		27 (4.6)	11 (4.3)	16 (4.8)	
Diabetes Mellitus		304 (51.4)	140 (54.3)	164 (49.3)	
Hypertension/Renal Vascular Disease		71 (12.0)	32 (12.4)	39 (11.7)	
Other		86 (14.6)	34 (13.2)	52 (15.6)	
Unknown/Missing		103 (17.4)	41 (15.9)	62 (18.6)	
**Cause of Death (Primary), n (%)**	517				0.71
Heart Disease		107 (20.7)	49 (20.1)	58 (21.3)	
Infectious Disease		34 (6.6)	13 (5.3)	21 (7.7)	
Diabetes Mellitus		77 (14.9)	33 (13.5)	44 (16.1)	
Cerebrovascular Accident		12 (2.3)	5 (2.1)	7 (2.6)	
Renal Disease		99 (19.2)	50 (20.5)	49 (18.0)	
Other		188 (36.4)	94 (38.5)	94 (34.4)	

**Cause of Death (Multiple), n (%)**					
Heart Disease	517	279 (54.0)	131 (53.7)	148 (54.2)	0.91
Infectious Disease	517	169 (32.7)	78 (32.0)	91 (33.3)	0.74
Diabetes Mellitus	517	130 (25.2)	59 (24.2)	71 (26.0)	0.63
Cerebrovascular Accident	517	37 (7.2)	16 (6.6)	21 (7.7)	0.62
Renal Disease	517	348 (67.3)	174 (71.3)	174 (63.7)	0.07

*For switchers, follow-up began at the date of the qualifying treatment change. For non-switchers, follow-up began at their dialysis initiation date plus the number of days that had elapsed from the switcher's initiation until the treatment change.

[a]The "Others" category encompasses individuals who do not identify as Jewish or Arab. This includes non-Arab Christians, members of other religions, and individuals without a religious classification.

P-values with bold font indicate statistical significance ($p<0.05$).
