# Supplementary material for: Outcomes of Dialysis Modality Switch: A Matched Cohort Analysis from a National Renal Replacement Therapy Registry, 2010–2022
Source: J Clin Med. 2026 May 20;15(10):3948. doi: 10.3390/jcm15103948 (PMC13207222; doi:10.3390/jcm15103948)
Supplement: Supplementary file 1 [file jcm-15-03948-s001.zip › Supp. Table S5 Switch 19.4.pdf]

Supplementary Table S5. Unadjusted and Adjusted Odds Ratios for Cause-Specific Death Within Two Years of Follow-Up Among Dialysis Modality Switchers Compared With Non-Switchers

**Outcome: Causes of Death (Within two years from follow-up)**	**Unadjusted Odds Ratio (95% Confidence Interval)**	**P-value**	**Adjusted Odds Ratio (95% Confidence Interval)**	**P-value**
Main Cause of Death				
All[a]				
Heart Disease	0.931 (0.608, 1.427)	0.74	0.944 (0.604, 1.474)	0.80
Infectious Disease	0.675 (0.331, 1.380)	0.28	0.655 (0.315, 1.365)	0.26
Diabetes Mellitus	0.814 (0.499, 1.327)	0.41	0.780 (0.471, 1.292)	0.34
Cerebrovascular Accident	0.795 (0.249, 2.538)	0.70	0.789 (0.237, 2.625)	0.70
Renal Disease	1.178 (0.760, 1.826)	0.46	1.221 (0.773, 1.931)	0.39
Other	1.193 (0.833, 1.709)	0.33	1.185 (0.817, 1.718)	0.37
Hemodialysis[b]				
Heart Disease	1.175 (0.555, 2.487)	0.67	1.214 (0.538, 2.739)	0.64
Infectious Disease	0.411 (0.116, 1.458)	0.17	0.332 (0.088, 1.256)	0.10
Diabetes Mellitus	0.883 (0.373, 2.092)	0.78	0.840 (0.338, 2.091)	0.71
Cerebrovascular Accident	1.144 (0.186, 7.013)	0.88	1.548 (0.217, 11.063)	0.66
Renal Disease	1.585 (0.734, 3.421)	0.24	1.592 (0.699, 3.627)	0.27
Other	0.871 (0.480, 1.582)	0.65	0.948 (0.508, 1.769)	0.87
Peritoneal Dialysis[b]				
Heart Disease	0.852 (0.500, 1.453)	0.56	0.852 (0.490, 1.480)	0.57
Infectious Disease	0.883 (0.371, 2.102)	0.78	0.900 (0.365, 2.216)	0.82
Diabetes Mellitus	0.813 (0.445, 1.487)	0.50	0.732 (0.391, 1.371)	0.33
Cerebrovascular Accident	0.548 (0.105, 2.865)	0.48	0.562 (0.096, 3.303)	0.52
Renal Disease	1.014 (0.584, 1.761)	0.96	1.078 (0.605, 1.919)	0.80
Other	1.369 (0.868, 2.159)	0.18	1.408 (0.877, 2.261)	0.16

Late switch[a]				
Heart Disease	0.820 (0.494, 1.363)	0.44	0.815 (0.480, 1.385)	0.45
Infectious Disease	0.681 (0.294, 1.577)	0.37	0.627 (0.265, 1.485)	0.29
Diabetes Mellitus	0.950 (0.549, 1.645)	0.85	0.854 (0.483, 1.510)	0.59
Cerebrovascular Accident	0.517 (0.106, 2.521)	0.41	0.625 (0.121, 3.239)	0.58
Renal Disease	1.250 (0.760, 2.058)	0.38	1.322 (0.788, 2.218)	0.29
Other	1.180 (0.780, 1.785)	0.43	1.250 (0.815, 1.917)	0.31
Early Switch[a]				
Heart Disease	1.117 (0.639, 1.952)	0.70	1.364 (0.725, 2.569)	0.34
Infectious Disease	0.667 (0.244, 1.820)	0.43	0.596 (0.204, 1.745)	0.35
Diabetes Mellitus	0.612 (0.295, 1.271)	0.19	0.613 (0.278, 1.351)	0.23
Cerebrovascular Accident	1.239 (0.314, 4.892)	0.76	1.146 (0.242, 5.441)	0.86
Renal Disease	1.069 (0.587, 1.945)	0.83	1.087 (0.558, 2.117)	0.81
Other	1.215 (0.750, 1.967)	0.43	1.061 (0.626, 1.800)	0.83
Hemodialysis + Late Switch[b]				
Heart Disease	1.238 (0.469, 3.267)	0.67	1.464 (0.465, 4.606)	0.51
Infectious Disease	0.278 (0.033, 2.345)	0.24	0.171 (0.018, 1.659)	0.13
Diabetes Mellitus	0.937 (0.301, 2.917)	0.91	0.716 (0.203, 2.530)	0.60
Cerebrovascular Accident	1.041 (0.091, 11.846)	0.97	2.068 (0.123, 34.845)	0.61
Renal Disease	1.733 (0.658, 4.561)	0.27	2.265 (0.763, 6.724)	0.14
Other	0.813 (0.367, 1.803)	0.61	0.882 (0.371, 2.095)	0.78
Hemodialysis + Early Switch[b]				
Heart Disease	1.139 (0.493, 2.631)	0.76	1.142 (0.456, 2.860)	0.78
Infectious Disease	0.490 (0.121, 1.975)	0.32	0.481 (0.116, 2.003)	0.31
Diabetes Mellitus	0.853 (0.321, 2.262)	0.75	0.850 (0.306, 2.362)	0.76
Cerebrovascular Accident	1.203 (0.165, 8.782)	0.86	1.242 (0.126, 12.279)	0.85
Renal Disease	1.503 (0.641, 3.524)	0.35	1.482 (0.600, 3.660)	0.39
Other	0.906 (0.465, 1.764)	0.77	0.980 (0.487, 1.970)	0.95
Peritoneal Dialysis + Late Switch[b]				

Heart Disease	0.704 (0.387, 1.280)	0.25	0.669 (0.358, 1.247)	0.21
Infectious Disease	0.865 (0.338, 2.213)	0.76	0.781 (0.293, 2.084)	0.62
Diabetes Mellitus	0.944 (0.504, 1.770)	0.86	0.884 (0.456, 1.711)	0.71
Cerebrovascular Accident	0.344 (0.040, 2.979)	0.33	0.519 (0.050, 5.368)	0.58
Renal Disease	1.109 (0.620, 1.985)	0.73	1.211 (0.656, 2.235)	0.54
Other	1.379 (0.847, 2.245)	0.20	1.476 (0.888, 2.453)	0.13
Peritoneal Dialysis + Early Switch[b]				
Heart Disease	1.534 (0.652, 3.610)	0.33	1.898 (0.768, 4.691)	0.16
Infectious Disease	0.952 (0.205, 4.424)	0.95	0.777 (0.150, 4.017)	0.76
Diabetes Mellitus	0.361 (0.082, 1.595)	0.18	0.293 (0.063, 1.360)	0.12
Cerebrovascular Accident	1.351 (0.152, 11.984)	0.79	0.540 (0.037, 7.788)	0.65
Renal Disease	0.679 (0.223, 2.070)	0.50	0.629 (0.193, 2.053)	0.44
Other	1.332 (0.593, 2.992)	0.49	1.372 (0.584, 3.227)	0.47
Multiple Causes of Death				
All[a]				
Heart Disease	0.979 (0.692, 1.384)	0.91	1.047 (0.730, 1.503)	0.80
Infectious Disease	0.940 (0.650, 1.358)	0.74	0.831 (0.565, 1.223)	0.35
Diabetes Mellitus	0.907 (0.609, 1.352)	0.63	0.813 (0.535, 1.235)	0.33
Cerebrovascular Accident	0.842 (0.429, 1.653)	0.62	0.847 (0.424, 1.695)	0.64
Renal Disease	1.414 (0.976, 2.050)	0.07	1.338 (0.910, 1.966)	0.14
Hemodialysis[b]				
Heart Disease	1.411 (0.784, 2.540)	0.25	1.370 (0.729, 2.572)	0.33
Infectious Disease	0.865 (0.471, 1.588)	0.64	0.750 (0.378, 1.486)	0.41
Diabetes Mellitus	1.072 (0.537, 2.142)	0.84	0.980 (0.465, 2.064)	0.96
Cerebrovascular Accident	0.906 (0.266, 3.083)	0.87	0.935 (0.261, 3.348)	0.92
Renal Disease	1.933 (1.006, 3.716)	**0.048**	1.784 (0.888, 3.584)	0.10
Peritoneal Dialysis[b]				
Heart Disease	0.851 (0.549, 1.319)	0.47	0.867 (0.549, 1.367)	0.54
Infectious Disease	0.934 (0.583, 1.498)	0.78	0.900 (0.548, 1.478)	0.68

Diabetes Mellitus	0.853 (0.518, 1.405)	0.53	0.727 (0.430, 1.228)	0.23
Cerebrovascular Accident	0.856 (0.376, 1.947)	0.71	0.839 (0.357, 1.969)	0.69
Renal Disease	1.142 (0.724, 1.803)	0.57	1.180 (0.736, 1.890)	0.49
Late switch[a]				
Heart Disease	0.811 (0.544, 1.210)	0.31	0.803 (0.528, 1.221)	0.30
Infectious Disease	0.980 (0.641, 1.498)	0.93	0.854 (0.546, 1.336)	0.49
Diabetes Mellitus	0.906 (0.571, 1.439)	0.68	0.748 (0.460, 1.216)	0.24
Cerebrovascular Accident	0.863 (0.395, 1.885)	0.71	0.837 (0.374, 1.872)	0.66
Renal Disease	1.403 (0.911, 2.160)	0.12	1.472 (0.937, 2.312)	0.09
Early Switch[a]				
Heart Disease	1.324 (0.822, 2.131)	0.25	1.765 (1.032, 3.019)	**0.038**
Infectious Disease	0.879 (0.531, 1.455)	0.62	0.698 (0.397, 1.228)	0.21
Diabetes Mellitus	0.909 (0.529, 1.562)	0.73	0.999 (0.546, 1.826)	1.00
Cerebrovascular Accident	0.809 (0.316, 2.069)	0.66	0.905 (0.325, 2.524)	0.85
Renal Disease	1.433 (0.861, 2.385)	0.17	1.360 (0.778, 2.378)	0.28
Hemodialysis + Late Switch[b]				
Heart Disease	0.914 (0.418, 1.999)	0.82	0.881 (0.363, 2.141)	0.78
Infectious Disease	0.849 (0.378, 1.909)	0.69	0.527 (0.197, 1.404)	0.20
Diabetes Mellitus	1.210 (0.495, 2.957)	0.68	0.835 (0.299, 2.335)	0.73
Cerebrovascular Accident	0.822 (0.152, 4.445)	0.82	0.716 (0.116, 4.412)	0.72
Renal Disease	3.623 (1.271, 10.328)	**0.016**	3.645 (1.154, 11.511)	**0.028**
Hemodialysis + Early Switch[b]				
Heart Disease	1.816 (0.937, 3.519)	0.08	1.716 (0.835, 3.528)	0.14
Infectious Disease	0.874 (0.442, 1.725)	0.70	0.860 (0.400, 1.852)	0.70
Diabetes Mellitus	0.997 (0.457, 2.174)	0.99	1.046 (0.453, 2.416)	0.92
Cerebrovascular Accident	0.955 (0.246, 3.711)	0.95	0.880 (0.203, 3.821)	0.86
Renal Disease	1.464 (0.719, 2.982)	0.29	1.380 (0.646, 2.948)	0.41
Peritoneal Dialysis + Late Switch[b]				
Heart Disease	0.757 (0.473, 1.209)	0.24	0.730 (0.445, 1.199)	0.21

Infectious Disease	1.047 (0.635, 1.725)	0.86	0.993 (0.584, 1.687)	0.98
Diabetes Mellitus	0.814 (0.474, 1.398)	0.46	0.701 (0.395, 1.242)	0.22
Cerebrovascular Accident	0.864 (0.357, 2.089)	0.75	0.919 (0.363, 2.327)	0.86
Renal Disease	1.109 (0.680, 1.808)	0.68	1.207 (0.723, 2.014)	0.47
Peritoneal Dialysis + Early Switch[b]				
Heart Disease	1.362 (0.602, 3.083)	0.46	1.731 (0.726, 4.131)	0.22
Infectious Disease	0.569 (0.220, 1.468)	0.24	0.461 (0.167, 1.276)	0.14
Diabetes Mellitus	1.014 (0.423, 2.427)	0.98	0.863 (0.341, 2.189)	0.76
Cerebrovascular Accident	0.824 (0.179, 3.786)	0.80	0.597 (0.112, 3.178)	0.55
Renal Disease	1.283 (0.554, 2.969)	0.56	1.261 (0.528, 3.010)	0.60

Early switch was defined as switching within 180 days of treatment initiation; late switch was defined as switching more than 180 days after treatment initiation.

[a]Analyses were adjusted for age group, sex, population group, peripherality, first modality, and incident-year cohort.

[b]Analyses were adjusted for age group, sex, population group, peripherality, and incident-year cohort.

*N Observations used (Unadjusted, Adjusted):

All = **517, 516**

Hemodialysis = **183, 182**

Peritoneal Dialysis = **334, 334**

Late Switch = **422, 422**

Early Switch = **368, 367**

Hemodialysis + Late Switch = **117, 117**

Hemodialysis + Early Switch = **145, 144**

Peritoneal Dialysis + Late Switch = **305, 305**

Peritoneal Dialysis + Early Switch = **223, 223**
